# The mediation effect of perceived weight stigma in association between weight status and eating disturbances among university students: is there any gender difference?

**DOI:** 10.1186/s40337-022-00552-7

**Published:** 2022-02-22

**Authors:** Kamolthip Ruckwongpatr, Mohsen Saffari, Xavier C. C. Fung, Kerry S. O’Brien, Yen-Ling Chang, Yi-Ching Lin, Chung-Ying Lin, Jung-Sheng Chen, Janet D. Latner

**Affiliations:** 1 Institute of Allied Health Sciences, College of Medicine, National Cheng Kung University, 1 University Rd, Tainan, 701401 Taiwan; 2 Health Research Center, Life Style Institute, Baqiyatallah University of Medical Sciences, Tehran, Iran; 3 Health Education Department, Faculty of Health, Baqiyatallah University of Medical Sciences, Tehran, Iran; 4 Department of Rehabilitation Sciences, Faculty of Health and Social Sciences, The Hong Kong Polytechnic University, Hung Hom, Hong Kong; 5 School of Social Sciences, Faculty of Arts, Monash University, Melbourne, VIC Australia; 6 Department of Family Medicine, Cardinal Tien Hospital, New Taipei, Taiwan; 7 Department of Early Childhood and Family Education, National Taipei University of Education, No.134, Sec. 2, Heping E. Rd., Da-an District, Taipei City, 106 Taiwan; 8 Department of Occupational Therapy, College of Medicine, National Cheng Kung University, Tainan, 701401 Taiwan; 9 Biostatistics Consulting Center, National Cheng Kung University Hospital, College of Medicine, National Cheng Kung University, Tainan, 701401 Taiwan; 10 Department of Public Health, College of Medicine, National Cheng Kung University, Tainan, 701401 Taiwan; 11 Department of Medical Research, E-Da Hospital, No. 6, Yida Rd., Yanchao Dist., Kaohsiung, 82445 Taiwan; 12 Department of Psychology, University of Hawaii at Manoa, Honolulu, HI USA

**Keywords:** Eating behaviors, Stigma, Weight control, Asian, Young adults

## Abstract

**Background:**

The study aimed to examine the association between perceived weight stigma (PWS), weight status, and eating disturbances. We hypothesized that PWS would partially mediate the association between weight status and eating disturbances among university students.

**Methods:**

The study involved 705 undergraduate students (379 females and 326 males) recruited from Hong Kong and Taiwan Universities (399 Hong Kong; 306 Taiwan participants). Our sample was from one Hong Kong university (located in Kowloon) and five Taiwan universities (three located in Southern Taiwan, one located in Central Taiwan, and one located in North Taiwan). Participants’ mean age was 20.27 years (SD = 1.79). All participants completed a demographic information sheet, the Three-Factor Eating Questionnaire-18 (TFEQ-R18), and the PWS questionnaire. PROCESS macro models were used to analyze potential mediations.

**Results:**

We found a significantly higher PWS scores in a high weight group for females and males. There was a significant difference between weight status and eating disturbances. Moreover, PWS partially mediated the association between weight status and eating disturbances for both genders.

**Conclusions:**

PWS is associated with weight status and eating disturbances, making it an important target for health improvement among young adults. Further studies are needed to corroborate such associations in participants from other societies and cultures.

## Abstract (plain language summary)

Perceived weight stigma, weight status (e.g., low weight, average weight, and high weight) and eating disturbances have been found to have associations between each other. However, such research is needed among Asians, and the present study examined a mediation mechanism among the three factors (i.e., perceived weight stigma, weight status, and eating disturbances). Using data from 705 university students across two regions (Hong Kong and Taiwan), the present study revealed that weight status might be associated with perceived weight stigma, and perceived weight stigma might be associated with eating disturbances. Such findings were consistent across male and female university students. The present study’s findings underscore the importance of perceived weight stigma. Reducing perceived weight stigma may be an important strategy in developing health eating behaviors among university students.

## Background

Weight stigma plays an important role in the social aspects of body weight, including negative attitudes, beliefs, and behaviors [1]. Individuals across the weight spectrum may experience negative judgements from others related to their weight [2], such as biases pertaining to being inactive, not intelligent, and lacking self-discipline [3]. Previous studies have shown that both individuals with high weight (38%) and non-high weight (7.3%) experience weight stigma [2], with 29% of adolescents categorized as having high weight reporting having experienced weight stigma [4]. Weight stigma can be classified into three general forms: weight-related self-stigma (or internalized weight stigma), perceived weight stigma (PWS), and experienced weight stigma [5]. Weight-related self-stigma refers to the internalization of stigmatizing beliefs, the acceptance and endorsement of discrimination directed against their individual characteristics. PWS can be defined as fear of being discriminated against. Experienced stigma indicates that the individual has experienced discrimination directed against them [6].

In previous research, PWS has been used to refer to both perceived and experienced weight stigma, terms that have seldom been distinguished [5]. One systematic review and meta-analysis highlighted that PWS could refer to both perceived and experienced weight stigma, and literature seldom explicitly separates perceived and experienced weight stigma. However, weight-related self-stigma is distinct from the two other types of weight stigma, PWS and experienced stigma. Furthermore, perceived and experienced weight stigma could be categorized as ‘public stigma,’ a broad term for stigma [7]. Following the findings from a systematic review and meta-analysis [5, 7], we used PWS to indicate both perceived and experienced weight stigma in the present study.

PWS may occur when a stigmatized individual has an awareness of the stereotypes, prejudice, and discrimination associated with their stigmatized condition [2]. Weight stigma is associated with numerous adverse outcomes including poorer mental health, stress, low self-esteem, body dissatisfaction, increased levels of obesity, unhealthy eating, and eating disturbance [8, 9]; thus, weight stigma is important public health issue [10]. PWS presents a critical issue for healthcare professionals, given that the PWS has been shown to be associated with both health behaviors and psychological distress [2]. Therefore, we decided to investigate PWS and its associations with weight status and eating disturbances.

Several studies have found that weight status is strongly related to PWS in individuals with high and low weight [9, 11–13]. A body of evidence indicates that PWS may be a predisposing factor to threatened social identity, which consequently may result in increasing stress, negative emotions, and avoidance [14]. Moreover, PWS is associated with mental health symptoms including depression, anxiety, body dissatisfaction, low self-esteem, and suicidal thoughts [15]. Specifically, people who are low weight also may suffer from PWS and its associated negative consequences. That is, individuals with low weight may feel they are being judged based on negative beliefs such as physical inability or weakness [12, 13, 16]. However, there are few studies on the association between low weight and PWS [17].

Some research has found that PWS may be related to unhealthy eating, poor diet, and weight fluctuation [18]. Those with PWS are more likely to report eating disturbances, and vice versa [19]. Moreover, PWS may foster and perpetuate eating disturbances [20]. Similarly, emotional distress (e.g., depression) was associated with PWS and eating disturbances [21, 22]. Eating disturbances have been conceptualized as including three domains: cognitive restraint (the level of cognitive control in daily food intake) [2], uncontrolled eating (disinhibition or overeating), and emotional eating (eating in response to negative emotions) [2]. Numerous studies found different associations between PWS and eating disturbances [23, 24].

Eating disturbances have been rising dramatically, specifically in Asian countries. This rise may be associated with cultural transition (i.e., Westernization) and is also related to gender [2, 23–25] and age. Chinese women of younger ages are at greater risk of eating disorders due to cultural values; they are encouraged to achieve thinness and avoid weight gain [26]. In contrast, weight gain in Chinese men is more socially accepted than in women [2]. Therefore, gender differences might be a determinative factor for eating disturbances and might be related to PWS. Thus, the associations between weight status, PWS, and eating disturbance are likely to be different between males and females.

Previous research found that PWS was different between females and males, suggesting that gender could be a potential moderator in the association between weight status and PWS [27]. Additionally, literature highlighted that females and males could have different eating disturbances that are influenced by different factors [28]. Thus, these different PWS levels may have differential associations with eating disturbances between females and males.

**Fig. 1:**
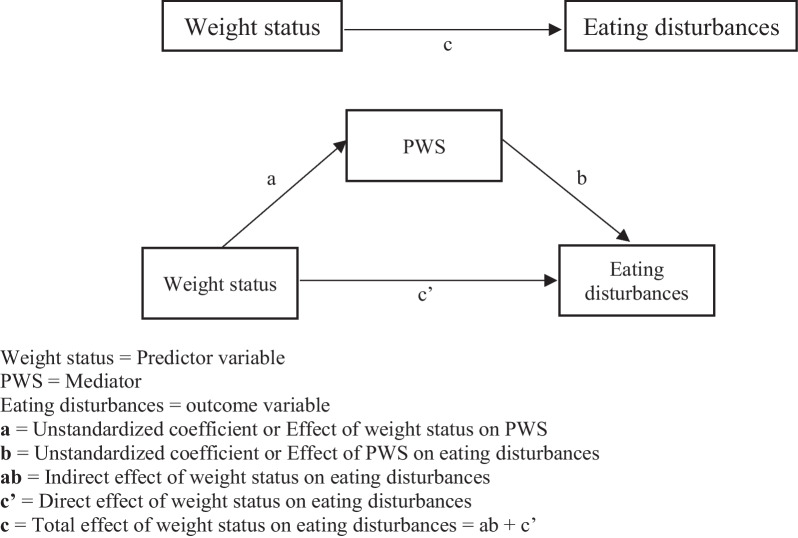
Hypotheses mediation model for weight status, PWS, and eating disturbances

Although previous studies have investigated the associations between weight status, PWS and eating disturbances, they have been conducted in Western cultures [4]. Western research on the association between weight PWS and eating disturbances in university students did not consider gender differences [4]. Little is known about these associations in Asians when considering potential gender differences. This study aimed to examine the relationship between PWS and eating disturbances, taking into account potential gender differences, in two Asian regions (i.e., Hong Kong and Taiwan). We focused on Asian participants across Hong Kong and Taiwan because both regions share a similar Chinese culture but later developed into distinctive subcultures based on different colonization histories (Hong Kong used to be governed by the United Kingdom and Taiwan used to be governed by Japan) [29]. We hypothesized that (1) weight status would be significantly associated with PWS in both genders; (2) weight status would be significantly associated with eating disturbances in both genders; (3) PWS would significantly and partially mediate the association between weight status and eating disturbances in both genders (Fig. 1).

## Method

### Participants

A total of 705 undergraduate students (379 females and 326 males) were recruited from Hong Kong and Taiwan Universities (399 Hong Kong; 306 Taiwan participants). They were administered a demographics questionnaire assessing age, gender, chronic illness, major, year of study, and self-reported anthropometric information (i.e., weight and height), and the different scale questionnaires. Completion of the study questionnaires occurred at the end of their classroom lecture and took approximately 20 min. Our sample was from one Hong Kong university (located in Kowloon), and five Taiwan universities (three located in Southern Taiwan, one located in Central Taiwan, and one located in North Taiwan). The mean age was 20.27 ± 1.79 years (20.13 ± 2.01 years for females and 20.44 ± 1.47 for males).

### Procedure

The study information and participants’ rights and confidentiality were provided to participants by the teaching faculty before data collection. Approval of this study was obtained from the ethics committee at the Hong Kong Polytechnic University (IRB ref. SEARS20161214002). All participants signed a written informed consent to verify their willingness of participation. The inclusion criteria for eligible participants included (1) participant age between 18 and 30 years; (2) capable writing and reading Chinese; (3) enrollment at universities in Hong Kong and Taiwan.

### Instruments

#### Demographics

According to WHO (2004) norms for Asia [30], we calculated the body mass index (BMI) using the self-reported anthropometric information (i.e., height and weight) into three groups of weight status, where a BMI < 18.5 kg/m^2^ is defined as low weight, BMI = 18.5–23.0 kg/m^2^ as average weight, and BMI > 23.0 kg/m^2^ as high weight.

#### Three-factor eating questionnaire-18 (TFEQ-R18)

The TFEQ is a self-report questionnaire that investigates eating disturbances [31]. TFEQ-R18 consists of 18 items, including three domains: cognitive restraint, uncontrolled eating, and emotional eating [4]. A sample item of cognitive restraint is “I deliberately take small helpings as a means of controlling my weight.” A sample item of uncontrolled eating is “Sometimes when I start eating, I just can’t seem to stop.” A sample item of emotional eating is “When I feel blue, I often overeat.” The items of TFEQ are rated on a 4-point response scale (definitely true/mostly true/mostly false/definitely false) [4]. The three domains of TFEQ were converted to a scale from 0 to 100. Consequently, higher scores refer to a higher likelihood of cognitive restraint, uncontrolled eating, or emotional eating [4]. The internal consistency of the TFEQ was satisfactory (α = 0.78–0.87) [25] in the English version and in the Chinese version (α = 0.79–0.82) [25]. Additionally, the reliability of TFEQ was considered sufficient (composite reliability = 0.87–0.89; *α* = 0.86–0.89) [32]. Moreover, the internal consistency of TFEQ was satisfactory in the Hong Kong and Taiwan sample in this study (α = 0.82).

#### Perceived weight stigma questionnaire (PWS)

The PWS is a self-report scale that investigates perceptions of weight-based stigmatization experiences. The PWS consists of 10 dichotomously scored items (score 0 indicates no and score 1 indicates yes). An example item is “People behave as if you are inferior because of your weight status.” Responses were summed, and higher scores indicate greater perceived weight stigma. The internal consistency of the PWS was acceptable in the Chinese version (α = 0.84) [25] and in the Hong Kong and Taiwan sample in this study (α = 0.84). Moreover, PWS is a unidimensional factor structure with satisfactory fit indices, as demonstrated through confirmatory factor analysis across Hong Kong and Taiwan people [25].

### Statistical analysis

All data were analyzed using the SPSS statistics version 26 (IBM Corp., Armonk, NY). One-way analysis of variance (ANOVA) was performed for females and males to analyze differences in TFEQ (including total and three domain scores) and PWS between three weight groups (i.e., low, average, and high weight) and presenting in the bar graph. Pearson correlations were performed to analyze the association between PWS and TFEQ (including total and three domain scores). Additionally, Fisher Z tests was performed to calculate the differences in the females’ and males’ correlations.

Multiple linear regression models were performed separately for females and males to analyze how eating disturbance was associated with weight status and PWS. Specifically, we included weight status and PWS as the independent variables and TFEQ (including total scores and three domain scores) as dependent variable. In all the regression models, age, and chronic diseases were included as the controlled variables. However, our regression models include PWS as the dependent variable to investigate PWS as a mediator in the association between weight status and eating disturbances in the mediation analysis. Moreover, we treated the average weight as the reference group because we considered that participants of average weight have experienced less weight stigma than participants with low or high weights.

Additionally, mediation models were performed to analyze PWS as mediator of the association between weight status and TFEQ (including total and three domain scores); we separated the mediation model for males and females. The mediated effect and the 95% confidence intervals (CIs) were used to explain the significance of the association, where present. We considered weight status and PWS as independent variables and TFEQ (including total and three subscales score) as dependent variables. The mediation models were designed using Hayes’ Model 4 in the PROCESS macro via SPSS with 5000 bootstrapping resamples adopted [33].

## Results

Participants’ demographic information is presented in Table 1. We found that there were significant differences in BMI, major, and year of study between females and males. However, there were no significant differences in age or chronic illness between females and males. Results of gender differences between low, average, high weight in PWS and eating disturbances are presented in Table 2.

**Table 1:** Participants’ characteristic

Variables	Females		Males		t	*P* value
	Mean (SD)	n (%)	Mean (SD)	n (%)		
Age	20.13 (2.01)	381 (53.9)	20.44 (1.47)	326 (46.1)	− 2.28	0.023*
BMI	20.65 (2.97)		21.48 (3.55)		− 3.32	< .001*
Low weight	17.57 (0.75)	74 (19.4)	17.60 (0.78)	49 (15.0)		
Average weight	20.27 (1.14)	241 (63.3)	20.65 (1.26)	196 (60.1)		
High weight	25.67 (3.18)	64 (16.8)	25.83 (4.10)	81 (24.8)		
Missing		2 (0.5)		–		
Major					− 7.04	< .001*
Health and social sciences		206 (54.1)		94 (28.8)		
Others		175 (45.9)		232 (71.2)		
Year of study					− 4.76	< .001*
1st year		143 (37.5)		76 (23.3)		
2nd year		118 (31.0)		86 (26.4)		
3rd year		88 (23.1)		125 (38.3)		
4th year		14 (3.7)		30 (9.2)		
5th year		10 (2.6)		7 (2.1)		
6th year		2 (0.5)		–		
Missing		6 (1.6)		2 (0.6)		
Chronic illness					− 0.53	0.60
Yes	–	17 (4.5)		12 (3.7)		
No	–	363 (95.3)		314 (96.3)		

*p < .05

**Table 2:**
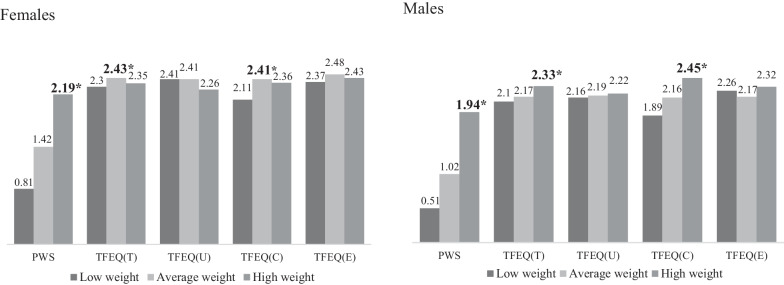
Bar graph presenting differences in PWS and TFEQ between low, average, and high weight groups

*PWS* perceived weight stigma, *TFEQ* three factor eating questionnaire, *TFEQ(T)* total scores, *TFEQ(U)* uncontrolled eating, *TFEQ(C)* cognitive restrained eating, *TFEQ(E)* emotional eating

*p < .05, Post hoc-Scheffe

As shown in Table 2, we found that females and males with high weight had significantly higher PWS scores than participants from the other weight categories. Moreover, males with high weight had significantly higher TFEQ (including total and cognitive subscale) scores than participants from the other weight categories. However, we found females with average weight had significantly higher TFEQ (including total and cognitive) scores than participants from the other weight categories.

### Correlation of PWS, TFEQ (total and three subscales) between females and males

As shown in “Appendix 1”, Fisher Z tests showed that there were no significant differences in the correlations between PWS and TFEQ (including total and three subscales) between females and males.

### Correlations between PWS and TFEQ (total and three subscales)

As shown in “Appendix 2”, Pearson correlations showed that TFEQ total scores were significantly and positively associated with PWS (r = 0.25; *p* < 0.001). Moreover, all subscale scores of TFEQ were significantly and positively associated with PWS; TFEQ (uncontrolled) scores (r = 0.16; *p* < 0.001), TFEQ (cognitive) scores (r = 0.18; *p* < 0.001), and TFEQ (emotional) scores (r = 0.20; *p* < 0.001).

### Regression models on TFEQ, PWS, low and high weight

As shown in Table 3, in female participants, after controlling for age and illness status, regression models showed that PWS significantly explained eating disturbances across all domains, including TFEQ total scores (beta = 0.04, *p* < 0.001); uncontrolled scores (beta = 0.04, *p* = 0.003); cognitive scores (beta = 0.04, *p* = 0.004); emotional scores (beta = 0.05, *p* = 0.002). Participants with low weight had significantly lower TFEQ total scores (beta = − 0.12, *p* < 0.02); cognitive scores (beta = − 0.29, *p* < 0.001) than those with average weight. Participants with high weight had significantly lower TFEQ total scores (beta = − 0.11, *p* = 0.037); uncontrolled scores (beta = − 0.19, *p* = 0.014). Furthermore, the low weight group had a significantly lower PWS scores (beta = − 0.64, *p* = 0.024) than did the average weight group. The participants with high weight had a significantly higher PWS scores (beta = 0.74, *p* = 0.013) than did the average weight group.

**Table 3:** Multiple linear regression models on Age, chronic illness, PWS, TFEQ, and BMI (low weight, high weight)

	B (SE)/*p* value				
	TFEQ(T)	TFEQ(U)	TFEQ(C)	TFEQ(E)	PWS
*Females*					
Age	− 0.01 (0.01)/0.434	− 0.01 (0.01)/0.349	− 0.003 (0.01)/0.818	− 0.01 (0.02)/0.679	− 0.01 (0.05)/0.813
Chronic illness (no)	− 0.02 (0.10)/0.879	0.01 (0.14)/0.951	− 0.18 (0.13)/0.177	0.13 (0.17)/0.471	− 1.33 (0.54)/0.014
PWS	0.04 (0.01)/< .001*	0.04 (0.01)/0.003*	0.04 (0.01)/0.004*	0.05 (0.02)/0.002*	–
Low weight (Ref)	− 0.12 (0.05)/0.020*	0.02 (0.07)/0.796	− 0.29 (0.07)/< .001*	− 0.09 (0.09)/0.322	− 0.64 (0.28)/0.024*
High weight (Ref)	− 0.11 (0.05)/0.037*	− 0.19 (0.08)/0.014*	− 0.08 (0.07)/0.257	− 0.07 (0.10)/0.468	0.74 (0.30)/0.013*
R^2^ (Adj. R^2^)	0.08 (0.06)	0.04 (0.02)	0.08 (0.07)	0.03 (0.02)	0.05 (0.04)
*Males*					
Age	0.002 (0.02)/0.902	− 0.02 (0.02)/0.439	0.02 (0.02)/0.421	0.004 (0.03)/0.866	− 0.09 (0.08)/0.223
Chronic illness (no)	− 0.04 (0.12)/0.734	− 0.26 (0.15)/0.082	0.03 (0.16)/0.852	0.11 (0.20)/0.579	− 0.04 (0.59)/0.946
PWS	0.05 (0.01)/< .001*	0.04 (0.01)/0.004*	0.03 (0.02)/0.059*	0.08 (0.02)/< .001*	–
Low weight (Ref)	− 0.05 (0.07)/0.469	− 0.01 (0.08)/0.910	− 0.25 (0.09)/0.004*	0.12 (0.11)/0.275	− 0.52 (0.32)/0.107
High weight (Ref)	0.11 (0.06)/0.047*	− 0.01 (0.07)/0.905	0.27 (0.07)/< .001*	0.07 (0.09)/0.429	0.94 (0.27)/< .001*
R^2^ (Adj. R^2^)	0.09 (0.07)	0.04 (0.02)	0.11 (0.10)	0.06 (0.04)	0.06 (0.05)

*Ref* reference** = **average weight, *PWS* perceived weight stigma, *TFEQ* three factor eating questionnaire, *TFEQ(T)* total scores, *TFEQ(U)* uncontrolled eating, *TFEQ(C)* cognitive restrained eating, *TFEQ(E)* emotional eating

*p < .05

In male participants, after controlling for age and illness status, the regression models showed that PWS significantly explained eating disturbances in all domains, including TFEQ total scores (beta = 0.05, *p* < 0.001); uncontrolled scores (beta = 0.04, *p* = 0.004); cognitive scores (beta = 0.03, *p* = 0.059); and emotion scores (beta = 0.08, *p* < 0.001). Participants with low weight had significantly lower TFEQ (cognitive) scores (beta = − 0.25, *p* = 0.004), and participants with high weight had significantly higher TFEQ including total scores (beta = 0.11, *p* = 0.047); cognitive scores (beta = 0.27, *p* < 0.001) than those with average weight. Furthermore, the participants with high weight had significantly higher PWS scores (beta = 0.94, *p* < 0.001) than did the average weight group.

### Mediation model of the effect of weight status on PWS, and TFEQ

**Fig. 2:**
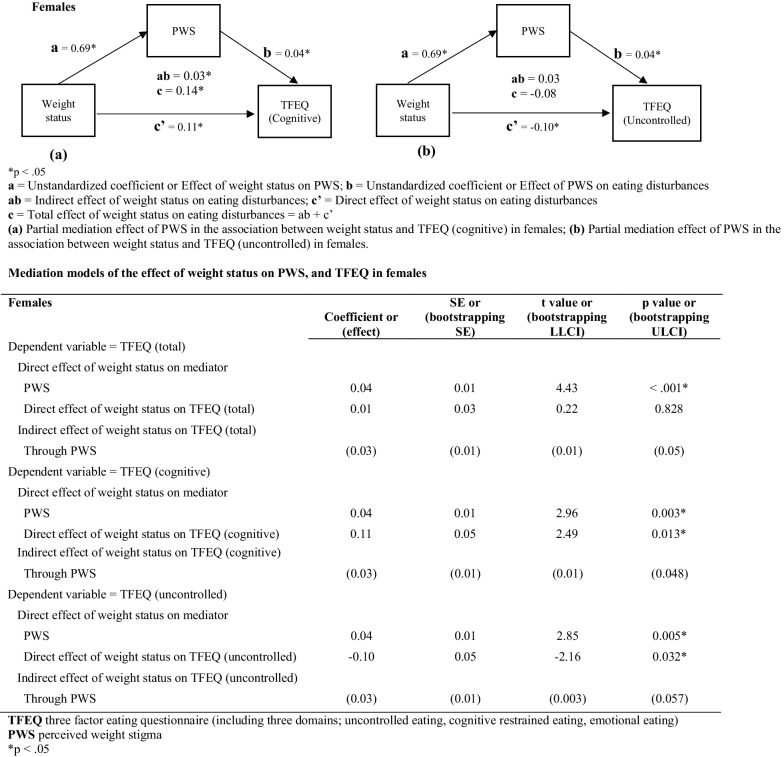
Mediation model for weight status, PWS, and eating disturbances in females

As shown in Fig. 2, in female participants, PWS significantly partially mediated the association between weight status and TFEQ. The total effect of weight status on PWS was 0.69 (SE = 0.18; t = 3.80; *p* = 0.0002). The total effect of PWS on TFEQ (total) was 0.04 (SE = 0.01; t = 4.43; *p* < 0.001). Therefore, PWS partially mediated the association between weight status and TFEQ (total scores) in female participants.

**Fig. 3:**
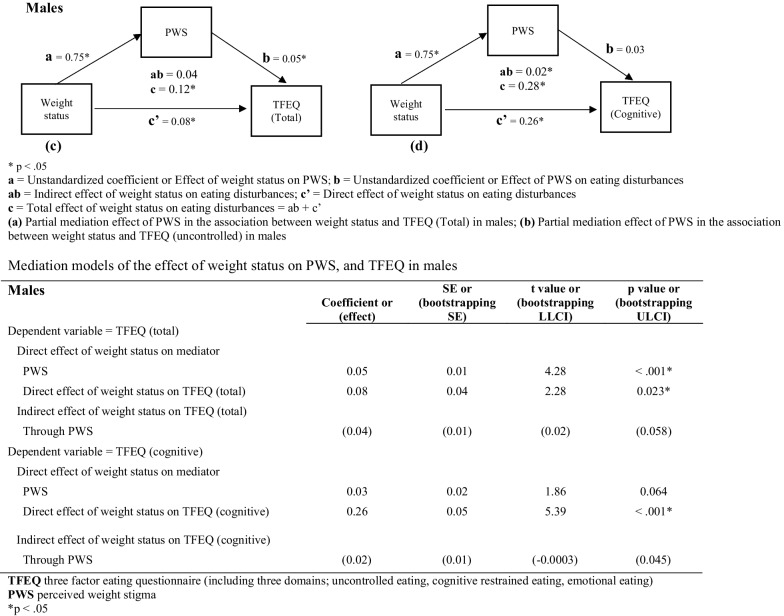
Mediation model for weight status, PWS, and eating disturbances in males

As shown in Fig. 3, in male participants, the total effect of weight status on PWS was 0.75 (SE = 0.18; t = 4.22; *p* < 0.001). The total effect of PWS on TFEQ (total) was 0.05 (SE = 0.01; t = 4.28; *p* < 0.001). PWS significantly partially mediated the association between weight status and TFEQ (total scores) in male participants. Additionally, the total effect of weight status on TFEQ (total) was 0.08 (SE = 0.04; t = 2.28; *p* = 0.023).

## Discussion

Our findings on the significant relationship between individuals with high weight and PWS were consistent with prior findings [4, 11, 25]. Young people with high weight may be suffering from prejudice and negative stereotype by their friends, educators, or parents during childhood, followed by increased vulnerability and sensitivity to PWS. Therefore, being overweight may increase risk of PWS and its negative consequences [34]. Previous research has demonstrated that individual with high weight could experience impaired social relationships associated with weight bias and greater PWS [34]. Apart from the associations between weight status and PWS, our findings were consistent with several studies reporting significant associations between PWS and eating disturbances [19, 20]. PWS may be associated with increased risk of eating pathology such as unhealthy weight control and uncontrolled eating and, in turn, may lead to weight gain [35]. However, this study also demonstrated that not all participants with PWS showed greater eating disturbances [35].

Consistent with a previous study [4], our study demonstrated a correlation between PWS and eating disturbances in low weight participants. Individuals with low weight could experience perceived pressure to be thin, which may be associated with body dissatisfaction or eating disturbances (i.e., cognitive restraint) [36]. Social and cultural pressure to achieve the thin ideal and fit ideal is associated with eating disturbances in young adults [26]. Specifically, in Chinese culture, young women highly value thinness [37].

Interestingly, we found that PWS was a partial mediator between weight status and eating disturbances. The mediating effect of PWS can be explained by the association between high weight and PWS [18]; and that of PWS and eating disturbances [35]. Specifically, individuals with high weight have experienced PWS and may become being vulnerable to failed dieting, eating disturbances, and weight fluctuation [18], because their PWS may increase their stress and lead to eating disturbances [37]. Previous research suggested that low weight was similarly associated with PWS, which led to eating disturbances [38]. Additionally, emotional distress (e.g., body dissatisfaction, lower self-esteem) could result from PWS and associated eating disturbances [35, 39]. Moreover, we found that weight status could lead to eating disturbances. Therefore, eating disturbances could be affected by weight status and partially mediated by PWS. However, we found the effect of weight status on PWS was large, while the effects of weight status and PWS on the TFEQ were small. This indicates that weight status may be an important variable in the contribution to PWS, while both weight status and PWS may be less important risk factors for eating disturbances.

Our results indicated that weight status was associated with PWS and eating disturbances in both men and women. Moreover, PWS mediated the relationship between weight status and eating disturbances in both men and women, similar to previous research [25]. Weight status, PWS, and eating disturbances were associated in males as well as females [40]. Males with high weight may engage in higher cognitive restrained eating than those in other weight categories, demonstrating that males with higher body weights may experience PWS and in turn use unhealthy weight-control behaviors as coping strategies [41]. However, we found that females with average weight demonstrated the highest scores in cognitive restrained eating and uncontrolled eating. One study highlighted that weight labels can significantly impact average weight individuals [42]. Many people of average weight might misperceive themselves as high weight, leading to greater body dissatisfaction and negative consequences [42, 43]. Moreover, the societal thin body ideal might create pressure to be thin and body dissatisfaction, followed by eating disturbances [23]. However, we collected our participants from across two regions (Hong Kong and Taiwan). We found that there were no differences between those two regions.

### Strengths and limitations

This study has several strengths. First, we focused on Asian participants across two regions (Hong Kong and Taiwan) which share a similar culture. Second, the mediation model found in the present study can explain that PWS could partially mediate the relationship between weight status and eating disturbances. There are also several limitations to the present study. First, we recruited participants using convenience sampling, so the generalizability of our findings may be limited. Second, this study was cross-sectional and thus cannot demonstrate causal relationships between variables. Third, we used self-reported questionnaires to collect all data, including the key variables in the study (i.e., anthropomorphic information, PWS, and eating disturbances). Findings from this type of data may be subject to recall biases, social desirability, and single-rater bias. However, the PWS and eating disturbances were assessed using validated instruments [2, 44] and the validity of self-reported height and weight has been previously found to be satisfactory [30]. Nevertheless, future studies are warranted using experimental or longitudinal designs with representative samples to corroborate our findings on the relationships between PWS and specific types of eating disturbances. Moreover, a study indicated that weight-related self-stigma could be more likely a mediator and moderator between PWS and eating disturbances [1]. Future studies should investigate whether weight-related self-stigma mediates or moderates in the association between PWS and eating disturbances to provide additional information and evidence in the weight bias field.

## Conclusion

PWS should be an important concern for healthcare providers because of its associations with weight status and eating disturbances. Moreover, we found that PWS played a mediational role between weight status and eating disturbances in both genders. Reducing PWS might improve eating disturbances. Future research should focus on investigating potential intervention strategies to increase awareness of PWS and to reduce its negative consequences.

## Data Availability

The datasets generated during and/or analyzed during the current study are available from the corresponding author on reasonable request.

## Appendix 1

See Table 4.

**Table 4:** Correlation of PWS, TFEQ (total and three subscales) between females and males

Variables		Overall	Females	Males	Gender differences in correlation	
					Z score	*P* value
PWS	TFEQ (total)	0.25**	0.23**	0.26**	− 0.59	0.56
PWS	TFEQ (uncontrolled)	0.16**	0.13*	0.17**	− 0.76	0.45
PWS	TFEQ (cognitive)	0.18**	0.18**	0.17**	0.19	0.85
PWS	TFEQ (emotional)	0.20**	0.16**	0.23**	− 1.35	0.18

*PWS* perceived weight stigma, *TFEQ* three factor eating questionnaire, *TFEQ(T)* total scores, *TFEQ(U)* uncontrolled eating, *TFEQ(C)* cognitive restrained eating, *TFEQ(E)* emotional eating; **p* < 0.05, ***p* < 0.01

## Appendix 2

See Table 5.

**Table 5:** Correlation between PWS and TFEQ (total and three subscales)

	TFEQ(T)	TFEQ(U)	TFEQ(C)	TFEQ(E)	PWS
TFEQ(T)	1				
TFEQ(U)	0.72**	1			
TFEQ(C)	0.52**	− 0.01	1		
TFEQ(E)	0.85**	0.53**	0.15**	1	
PWS	0.25**	0.16**	0.18**	0.20**	1

*PWS* perceived weight stigma, *TFEQ* three factor eating questionnaire, *TFEQ(T)* total scores, *TFEQ(U)* uncontrolled eating, *TFEQ(C)* cognitive restrained eating, *TFEQ(E)* emotional eating

***p* < 0.01

## Abbreviations

PWS: Perceived weight stigma
TFEQ-R18 or TFEQ: Three-factor eating questionnaire-18
BMI: Body mass index
ANOVA: Analysis of variance
CI: Confidence interval
