# Correction: The mediation effect of perceived weight stigma in association between weight status and eating disturbances among university students: is there any gender difference?

**DOI:** 10.1186/s40337-023-00842-8

**Published:** 2023-07-17

**Authors:** Kamolthip Ruckwongpatr, Mohsen Saffari, Xavier C. C. Fung, Kerry S. O’Brien, Yen-Ling Chang, Yi-Ching Lin, Chung-Ying Lin, Jung-Sheng Chen, Janet D. Latner

**Affiliations:** 1 Institute of Allied Health Sciences, College of Medicine, National Cheng Kung University, 1 University Rd, Tainan, 701401 Taiwan; 2 Health Research Center, Life Style Institute, Baqiyatallah University of Medical Sciences, Tehran, Iran; 3 Health Education Department, Faculty of Health, Baqiyatallah University of Medical Sciences, Tehran, Iran; 4 Department of Rehabilitation Sciences, Faculty of Health and Social Sciences, The Hong Kong Polytechnic University, Hung Hom, Hong Kong; 5 School of Social Sciences, Faculty of Arts, Monash University, Melbourne, VIC Australia; 6 Department of Family Medicine, Cardinal Tien Hospital, New Taipei, Taiwan; 7 Department of Early Childhood and Family Education, National Taipei University of Education, No.134, Sec. 2, Heping E. Rd., Da-an District, Taipei City, 106 Taiwan; 8 Department of Occupational Therapy, College of Medicine, National Cheng Kung University, Tainan, 701401 Taiwan; 9 Biostatistics Consulting Center, National Cheng Kung University Hospital, College of Medicine, National Cheng Kung University, Tainan, 701401 Taiwan; 10 Department of Public Health, College of Medicine, National Cheng Kung University, Tainan, 701401 Taiwan; 11 Department of Medical Research, E-Da Hospital, No. 6, Yida Rd., Yanchao Dist., Kaohsiung, 82445 Taiwan; 12 Department of Psychology, University of Hawaii at Manoa, Honolulu, HI USA

**Correction: Journal of Eating Disorders (2022) 10:28** 10.1186/s40337-022-00552-7

Following the publication of this article [1] we were informed that the first author's given and family names were inadvertently interchanged.

The author's given name is 'Kamolthip' and family name is 'Ruckwongpatr'.

The correct author name is included in the author list of this Correction. The original article has been revised.

